# Supplementary material for: Identification of Putative Novel Rotavirus H VP7, VP4, VP6 and NSP4 Genotypes in Pigs
Source: Viruses. 2023 Dec 30;16(1):68. doi: 10.3390/v16010068 (PMC10819321; doi:10.3390/v16010068)

Supplementary Figure S1. Similarity analysis of the VP4 gene of the strain ITA/14193-5/2020 and ITA/ 51105-6/2020 with RVH VP4 sequences. Strain name of RVH sequences are shown on the right of the plot. The analysis was performed by SimPlot 3.5.1 using a sliding window of 200 nucleotides moving in 20-bp steps.

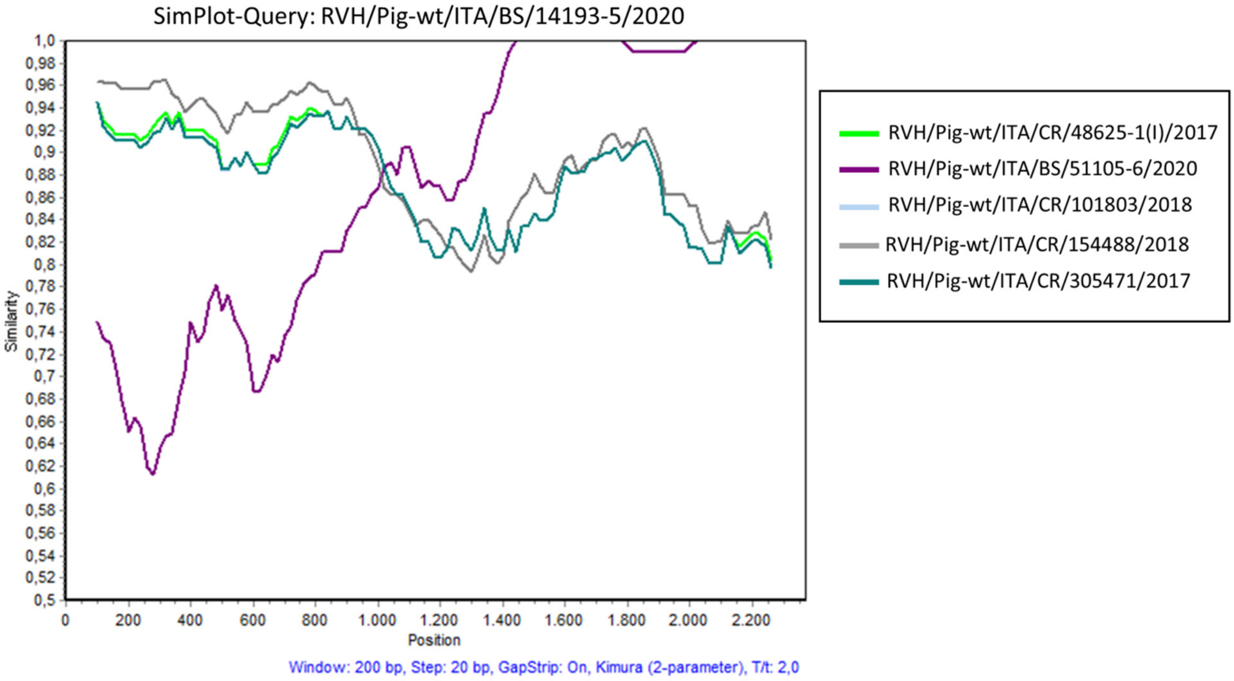

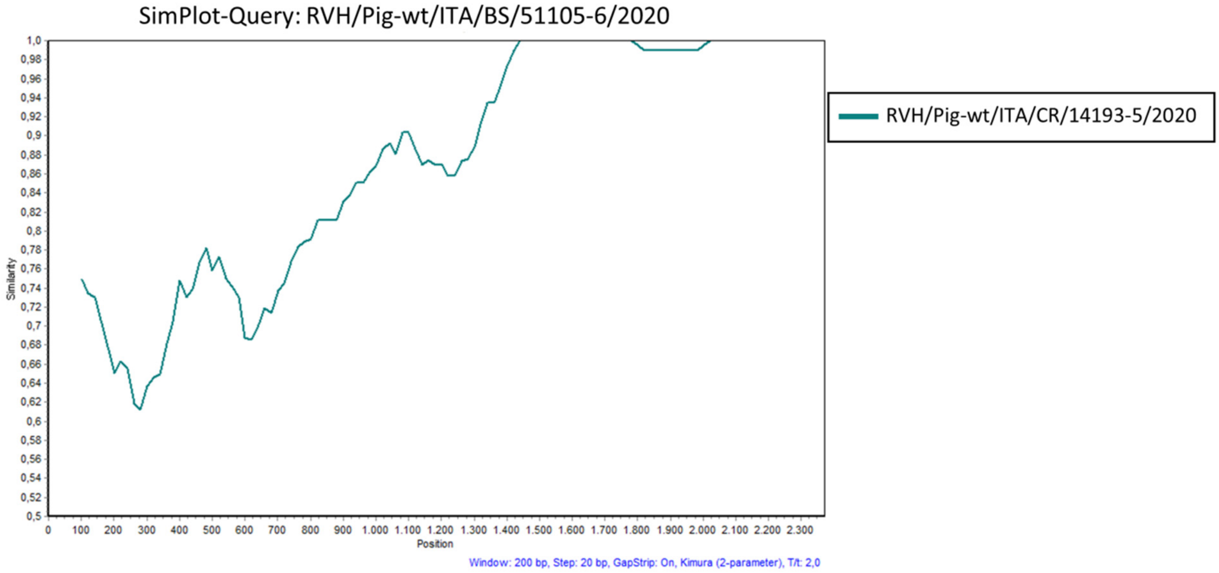

Supplement: Supplementary file 1 [file viruses-16-00068-s001.zip › Figure S1.pdf]
